# Analysis of an Educational Cathodic Protection System with a Single Drainage Point: Modeling and Experimental Validation in Aqueous Medium

**DOI:** 10.3390/ma11112099

**Published:** 2018-10-25

**Authors:** Luís Carlos Matos, José Inácio Martins

**Affiliations:** Faculdade de Engenharia, Universidade do Porto, Rua Dr. Roberto Frias, s/n 4200-465 Porto, Portugal; jipm@fe.up.pt

**Keywords:** cathodic protection, sacrificial anode, drainage point, attenuation curves

## Abstract

Cathodic protection, often taught in curricular units, such as corrosion and materials science, is an important subject in the study of chemical engineering. The implementation of lab setups and experimental activities in this field, are core to promoting understanding of the underlying concepts and to developing “hands-on” skills fundamental to the success of future process engineers. This paper reports the influence of different variables on the electrical potential and current behaviors of an educational cathodic protection system operated with a single drainage point. The system comprised a steel bar cathode connected to a zinc sacrificial anode, both placed in aqueous medium. The study variables were the anode area, the cathode diameter, the NaCl electrolyte concentration and the anode placement. Each variable showed a specific influence on the attenuation curves, allowing us to conclude that: (1) increasing the sacrificial anode area, or decreasing the resistivity of the medium, promotes more electronegative potentials on the structure and higher currents; (2) increasing the cathode diameter decreases the protection capacity; (3) positioning the anode in the middle of the cathode lengthwise gives rise to a more balanced potential distribution; and (4), the attenuation curves, both for potential and current, can be successfully predicted using equations based on Morgan and Uhlig’s work. High correlations were obtained between the experimental and modeling data for all the studied variables.

## 1. Introduction

Cathodic protection (CP) has been understood for about 170 years. It has primarily been used for protecting ordinary structural steel in soil and seawater, and less often for steel exposed to fresh water. The use of this technology has increased considerably in recent decades in response to expanding offshore oil and gas exploration and production. It is the main protection method for the large submerged parts of fixed oil installations, and is often the only protection for parts freely exposed to seawater [1].

The corrosion of iron in aqueous medium can be expressed by the following equations [2]:

(1) $$2\mathrm{Fe}\to 2{\mathrm{Fe}}^{2+}+4e^{-}\;(\mathrm{anodic}\;\mathrm{reaction})$$

(2) $$O_{2}+2H_{2}O+4e^{-}\to 4{\mathrm{OH}}^{-}\;(\mathrm{cathodic}\;\mathrm{reaction})$$

However, depending on the pH and oxygenation of the medium, the ferrous ions can give rise to passive films, which according to Gui and Devin [3] correspond to a mixture of Fe(OH)_2_ and γ − Fe_2_O_3_ /Fe_3_O_4_, known as rust. The reactions (Equations (3) and (4)) describe the formation of these passive films.

(3) $$\;\mathrm{Fe}+2H_{2}O\to \mathrm{Fe}{\left(\mathrm{OH}\right)}_{2}+2H^{+}+2e^{-}\;$$

(4) $$\;\mathrm{Fe}{\left(\mathrm{OH}\right)}_{2}\to \gamma -\mathrm{FeOOH}+H^{+}+e^{-}\;$$

In saline medium, the adsorption of chloride ion on the iron surface develops an intermediate complex [4] that leads to the dissolution of the passive film; i.e., the nucleation for pitting corrosion. The following reactions may elucidate this behavior:

(5) $$\;{\mathrm{Fe}(\mathrm{OH})}_{2}{+\;\mathrm{Cl}}^{-}\to {\;\mathrm{Fe}(\mathrm{OHCl})+\mathrm{OH}}^{-}\;$$

(6) $$\;{\mathrm{Fe}(\mathrm{OHCl})\;+\;\mathrm{nCl}}^{-}\to {\;[\mathrm{Fe}(\mathrm{OH})\mathrm{Cl}}_{\left(n+1\right)}{]}^{-n}\;$$

(7) $$\;{\mathrm{Fe}(\mathrm{OH})}_{3}{+\;\mathrm{Cl}}^{-}\to \;\mathrm{Fe}(\mathrm{OH}{)}_{2}{\mathrm{Cl}\;+\mathrm{OH}}^{-}\;$$

(8) $$\;\mathrm{Fe}(\mathrm{OH}{)}_{2}{\mathrm{Cl}\;+\;\mathrm{nCl}}^{-}\to \;[\mathrm{Fe}(\mathrm{OH}{)}_{2}{\mathrm{Cl}}_{\left(n+1\right)}{]}^{-n}\;$$

(9) $$\;{{[\mathrm{Fe}(\mathrm{OH})\mathrm{Cl}}_{\left(n+1\right)}]}^{-n}{\;+\;H}^{+}\to {\;H}_{2}{O\;+\;\mathrm{Fe}}^{2+}{\;+\;(n+1)\;\mathrm{Cl}}^{-}\;$$

(10) $$\;\mathrm{Fe}(\mathrm{OH}{)}_{2}{\mathrm{Cl}}_{\left(n+1\right)}{]}^{-n}{\;+\;2H}^{+}\to {\;2H}_{2}{O\;+\;\mathrm{Fe}}^{3+}{\;+\;(n+1)\;\mathrm{Cl}}^{-}\;$$

Cathodic protection is an electrochemical technique based on the application of a cathodic polarization to an engineering structure in order to prevent corrosion [5]. The simplest method of protecting the structure is to bind the structure to a more active metal, such as zinc, i.e., transferring the corrosion to that metal, using a sacrificial anode. In this case, instead of the occurrence of Equation (1), the following reaction takes place:

(11) $$\;2\mathrm{Zn}\to 2{\mathrm{Zn}}^{2+}+4e^{-}\;$$

Although the O_2_ reduction is considered to be the cathodic reaction predominant in alkaline and oxygenated media, the release of hydrogen may occur in deaerated alkaline or acidic media [6]:

(12) $$\;2H^{+}+2e^{-}\to H_{2}\;$$

(13) $$\;2H_{2}O+2e^{-}\to H_{2}++2{\mathrm{OH}}^{-}\;$$

**Figure 1:**
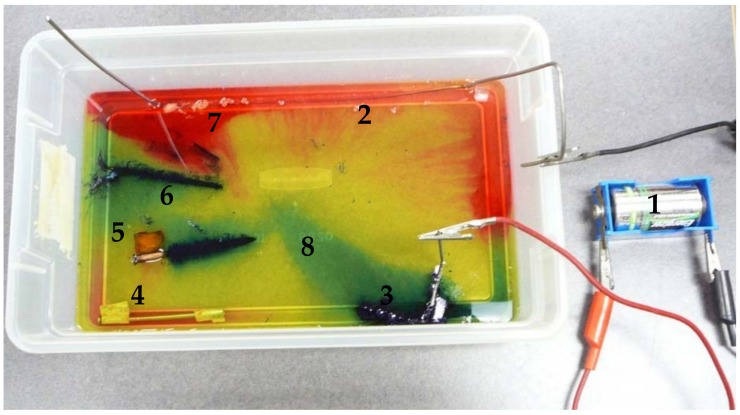
Illustration of corrosion and cathodic protection systems. Legend: 1. DC power supply for cathodic protection by impressed current; 2. iron structure to be protected; 3. scrap auxiliary anode; 4. cathodic protection by sacrificial zinc anode; 5. corrosion by galvanic coupling, Fe-Cu; 6. corrosion by local action cells; 7. cathodic areas, red color, reaction 2H_2_O + 2e → H_2_ + 2OH^−^ or O_2_ + 2H_2_O + 4e → 4OH^−^; 8. anodic areas, Prussian blue color due the interaction of Fe^2+^ with [Fe(CN)_6_]^3−^.

For long structures such as pipelines, where sacrificial anode protection becomes unsuitable, an external DC (direct current) power source is used to provide the required current. A didactic color-based experiment performed to illustrate the two cathodic protection processes is shown in Figure 1. The composition of the electrolyte (NaCl, K_3_Fe(CN)_6_, phenolphthalein and agar–agar) shows the reactions that take place in the system: the cathodic reaction, hydrogen release or oxygen reduction areas are red due to alkalinization of the medium; and the anodic reaction zones are blue due to the interaction of ferrous ions with ferricyanide ions.

**Figure 2:**
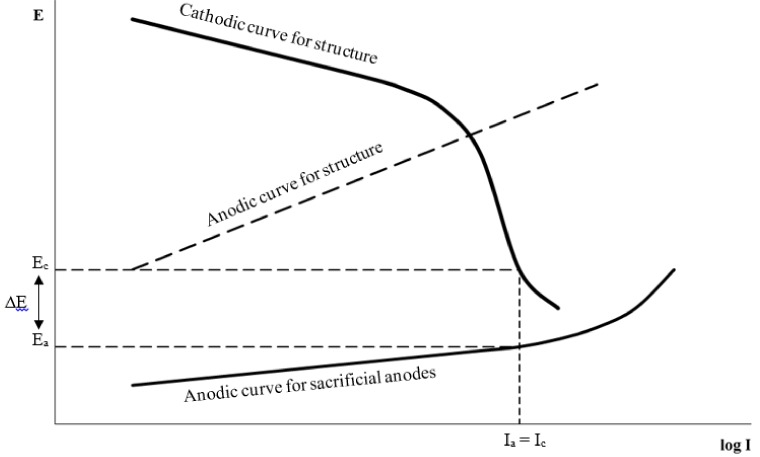
Evan’s diagram of a cathodic protection system with sacrificial anodes (adapted from Reference [1]).

**Figure 3:**
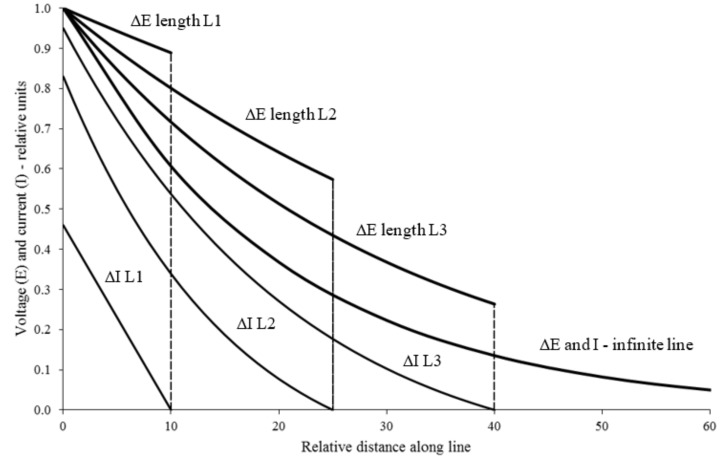
Attenuation curves (relative voltage and current) vs. distance to the draining point (adapted from Reference [8]).

The corrosion of any metal cannot take place if its surface potential in the electrolyte (*E*) is lower than the equilibrium potential for the oxidation process (*Eeq*). The condition in which the metal has no tendency to transit to the oxidized form (*E* ≤ *Eeq*) is called the “immunity” condition [7]. The Evan’s diagram shown in Figure 2 illustrates the potential-current profile in a cathodic protection system with sacrificial anodes. As has been referred to, the current required to force the electrode potential of the material to decrease can be created in two different ways; by means of a less noble material in the form of a sacrificial anode, or by means of an external current source, usually a rectifier [1]. Experience has shown that zinc is a good sacrificial anode in cathodic protection systems [4,5]. Current drained from a single point imposes a current density, and a potential, on the structure that decrease with distance from the draining point. The way they decrease is translated by an attenuation function that depends on many factors: medium resistivity, structure dimensions, and the presence of coatings, among others [6]. Figure 3 illustrates these attenuation curves in relative terms, correlating the voltage and current versus distance with the drainage point. Optimization studies showed a close relationship between the area and position of the anode and the potential needed to prevent the structure corrosion [7].

Morgan and Uhlig’s equations for predicting attenuation curves are well-known and have been described by several authors [5,9]. The following equation can be used to estimate the potential decrease along a structure [8,10,11]:

(14) $$E_{x}=E_{0}\exp\left(-\alpha x\right),$$

where *E_x_* is the potential at the position *x*, *E*_0_ is the potential when *x* = 0, the draining point, and *α* is the attenuation coefficient that can be calculated from Equation (15).

(15) $$\alpha =\sqrt{rk},$$

where *r* is the longitudinal resistance of the structure and *k* is the conductance per unit length.

The current intensity on the structure follows the same attenuation and can be estimated by the following equation:

(16) $$\;I_{x}=I_{0}\exp\left(-\alpha x\right)\;$$

The design of cathodic protection systems requires the calculation of the anode resistance, *R*. Several equations have been suggested according to anode geometry [1,12]; however, the following two equations are commonly used when the anode is positioned close to the surface:

(17) $$R=\frac{0.315\rho }{\sqrt{A}},$$

additionally known as the McCoy’s equation, where ρ is the medium resistivity and *A* is the anode area; and

(18) $$R=\frac{\rho }{2S},$$

where *S* is the arithmetic mean of the anode length and width.

In this work, one zinc sacrificial anode was connected to the structure and placed in two different drainage points for each set of runs; specifically, at the edge and in the middle of the cathode lengthwise. Two anodes with different areas were tested. Multiple experiments were performed under different conditions and the potential measurements were taken versus an Ag/AgCl reference electrode. The study variables were the anode area and placement, the cathode diameter and the NaCl concentration in aqueous solution. The attenuation predictions for both potential and current were obtained using Equations (14) and (16).

## 2. Materials and Methods

### 2.1. Cathodic Protection System with a Single Drainage Point

**Figure 4:**
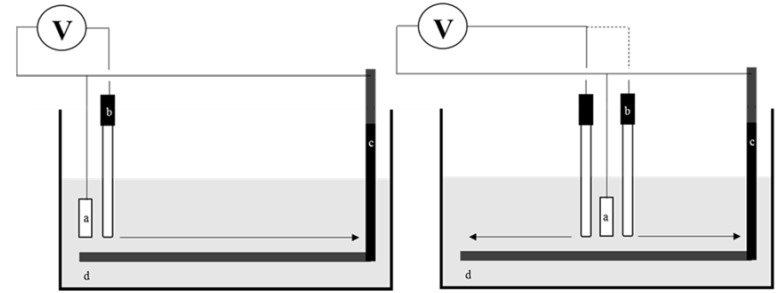
Experimental setup showing two configurations (sacrificial anode at the edge and in the middle of the cathode). Legend: (**a**) zinc sacrificial anode; (**b**) Ag/AgCl reference electrode; (**c**) cathode; (**d**) electrolyte.

The system comprised an acrylic vessel (0.4 m depth, 0.6 m length and 0.4 m high), a cylindrical steel cathode (0.47 m length with two possible diameters, 0.008 m and 0.015 m) and a rectangular zinc anode (two possible areas, 0.0018 m^2^ and 0.0058 m^2^). The Ag/AgCl reference electrode was held on an adjustable bracelet structure moving along a ruler. About 18 liters of NaCl solution was added into the vessel (two concentrations, 2.5% and 1.25% w/w, both with a pH of 7.5). The cathode was fixed to the vessel and connected to the sacrificial anode by electrical cables and crocodile clips. A sketch of the experimental setup is shown in Figure 4.

### 2.2. Instrumentation

The potential measurements along the structure were made in parallel against the Ag/AgCl reference electrode connected to a Protek 506 digital multimeter, while the current intensity measurements used the multimeter configured in series between the anode and cathode. The pH measurements were performed with a Symphony SP70P pH-meter, and the electrolyte resistivity with an EDT Re387Tx conductivity meter.

## 3. Results and Discussion

**Figure 5:**
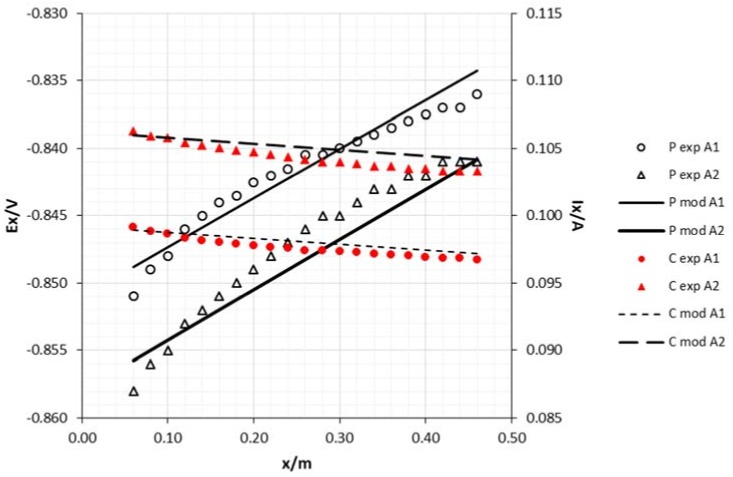
Experimental and model attenuation curves as a function of sacrificial anode area (cathode diameter 15 mm and edge draining point). Legend: P—potential; C—current; exp—experimental; mod—model; A1—anode area 0.0018 m^2^; A2—anode area 0.0054 m^2^.

**Figure 6:**
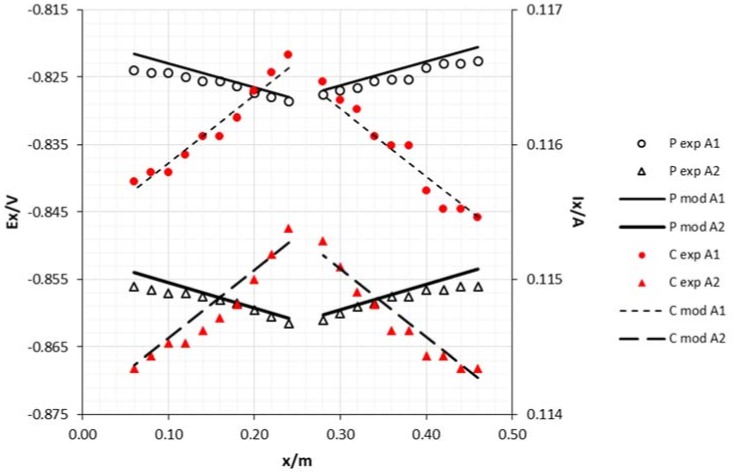
Experimental and model attenuation curves as a function of sacrificial anode area (cathode diameter 15 mm and middle draining point). Legend: P—potential; C—current; exp—experimental; mod—model; A1—anode area 0.0018 m^2^; A2—anode area 0.0054 m^2^.

The attenuation curves, for both potential and current intensity as a function of the sacrificial anode area are shown in Figure 5 and Figure 6. As can be seen, the increase of the anode area had a significant effect on the potential, making the steel structure more electronegative, thus increasing protection against corrosion. As was also expected, the current increased with the anode area. Some researchers have shown that steel structures in seawater are protected under potentials around −0.850 V vs. Ag/AgCl reference electrode [13], while other authors refer to −0.800 V as the recommended protective potential [1]. In fact, in all trials, the potentials were below this last reference.

The positioning of the sacrificial anode has a significant effect on the potential and current decreases. By positioning the anode in the middle of the cathode lengthwise, the decrease was smaller than when it was positioned at the edge. When the draining point is at the edge, the vessel wall becomes an obstacle to the current lines that just fall over one limited area. From a middle draining point, the current lines are symmetrically distributed along the length of the cathode, thus creating a more balanced potential and current.

**Figure 7:**
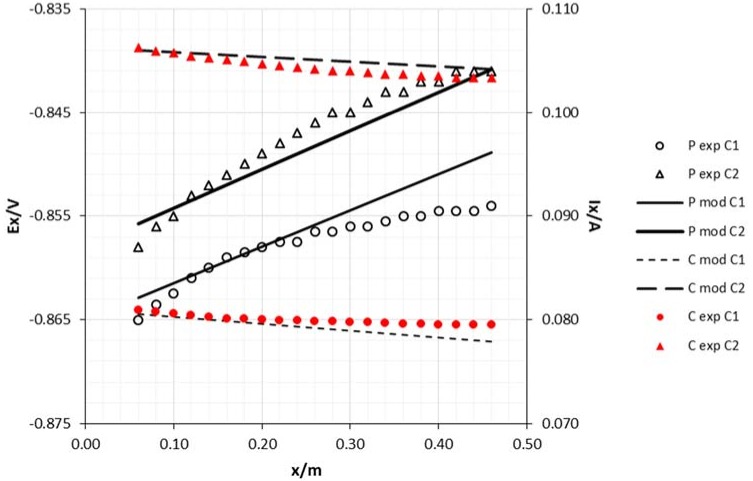
Experimental and model attenuation curves as a function of cathode area (sacrificial anode area: 0.0054 m^2^ and edge draining point). Legend: P—potential; C—current; exp—experimental; mod—model; C1—cathode diameter 8 mm; C2—cathode diameter 15 mm.

**Figure 8:**
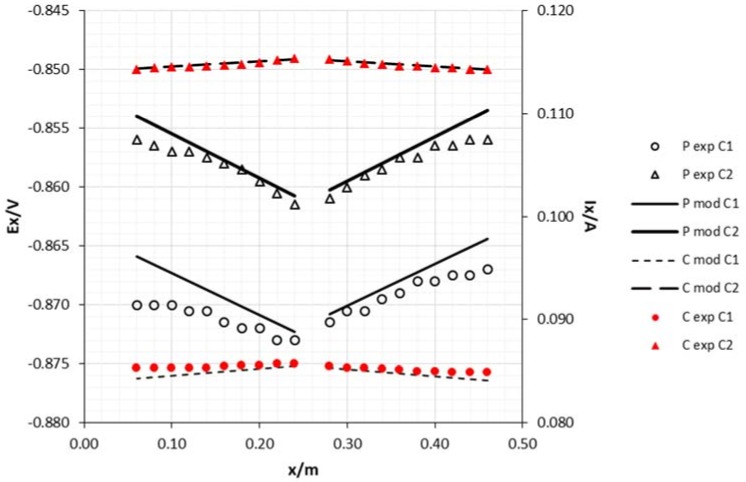
Experimental and model attenuation curves as a function of cathode area (sacrificial anode area: 0.0054 m^2^ and middle draining point). Legend: P—potential; C—current; exp—experimental; mod—model; C1—cathode diameter 8 mm; C2—cathode diameter 15 mm.

As can be seen in Figure 7 and Figure 8, the achieved potentials in the 8 mm diameter cathode are more electronegative than in the 15 mm rod. Meanwhile, the current has an opposite behavior which may be attributed to the slightly higher resistance of this rod according to Ohm’s Law.

**Figure 9:**
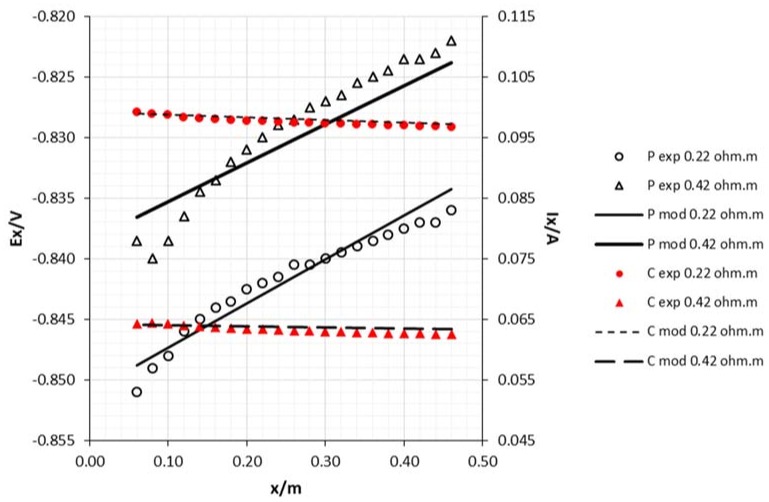
Experimental and model attenuation curves as a function of medium resistivity (sacrificial anode area: 0.0018 m^2^; cathode diameter: 15 mm and edge draining point). Legend: P—potential; C—current; exp—experimental; mod—model.

**Figure 10:**
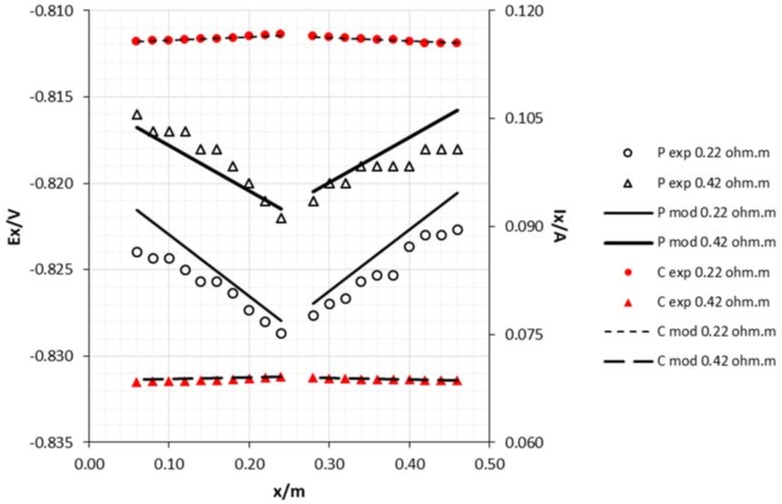
Experimental and model attenuation curves as a function of medium resistivity (sacrificial anode area: 0.0018 m^2^; cathode diameter: 15 mm and middle draining point). Legend: P—potential; C—current; exp—experimental; mod—model.

The resistivity of the medium plays an important role in cathodic protection [13,14,15]. As Figure 9 and Figure 10 show, the lower the resistivity, the more negative the potential, and the greater current in the structure.

Although the results presented in Figure 5, Figure 6, Figure 7, Figure 8, Figure 9 and Figure 10, clearly express a high correlation between experimental and modeling data, these values were subjected to Pearson’s correlation analysis using Statistica for Windows release 7.0, and the outcome is shown in Table 1 (correlation coefficients with *p* < 0.001).

## 4. Conclusions

Taking the experimental results into consideration, it is possible to draw the following conclusions:

1. Increasing the sacrificial anode area or decreasing the resistivity of the medium promotes more electronegative potentials on the structure and higher currents.
2. Increasing the cathode diameter decreases the protection capacity.
3. Positioning the anode in the middle of the cathode lengthwise, gives rise to a more balanced potential distribution.
4. Considering the experimental conditions described in this work, the attenuation curves, both for potential and current, can be successfully predicted using equations described in the literature.

These results, obtained from the study of technical details related to a cathodic protection project, enable a better practical understanding and theoretical articulation of core subjects in electrochemistry and corrosion science. Implementation of this educational setup in the Materials Science curricular unit of the Chemical Engineering course in the Faculty of Engineering at the University of Porto, would contribute to the development of “hands-on” skills fundamental to the success of future process engineers.

## Figures and Tables

**Table 1:** Correlation coefficients between experimental and model data for all studied variables.

Variable	Edge Draining Point		Middle Draining Point	
	Potential	Current	Potential	Current
Anode area 1 ^1^	0.9712	0.9712	0.9798	0.9794
Anode area 2 ^1^	0.9794	0.9794	0.9696	0.9682
Cathode area 1	0.9473	0.9480	0.9038	0.8616
Cathode area 2	0.9794	0.9794	0.9696	0.9682
Resistivity 1	0.9712	0.9712	0.9798	0.9794
Resistivity 2	0.9798	0.9797	0.7840	0.7196

^1^ 1 and 2 indices correspond to the anode and cathode areas, as well as to the resistivity mentioned in Figure 5, Figure 6, Figure 7, Figure 8, Figure 9 and Figure 10.
